# Supplementary material for: Evidence of thermophilization in Afromontane forests
Source: Nat Commun. 2024 Jul 10;15:5554. doi: 10.1038/s41467-024-48520-w (PMC11236992; doi:10.1038/s41467-024-48520-w)
Supplement: Supplementary file 1 — Supplementary Information [file 41467_2024_48520_MOESM1_ESM.pdf]

Supplementary Information

Evidence of thermophilization in Afromontane forests

Aida Cuni-Sanchez[1,2], Emanuel H. Martin[3], Eustrate Uzabaho[4], Alain S. K. Ngute[5], Robert Bitariho[6], Charles Kayijamahe[4], Andrew R. Marshall[5,7], Nassoro A. Mohamed[3], Gideon A. Mseja[3], Aventino Nkwasibwe[6], Francesco Rovero[8,9], Douglas Sheil[10], Rogers Tinkasimire[6], Lawrence Tumugabirwe[6], Kenneth J. Feeley[11,12], Martin J. P. Sullivan[13]

Supplementary Figures

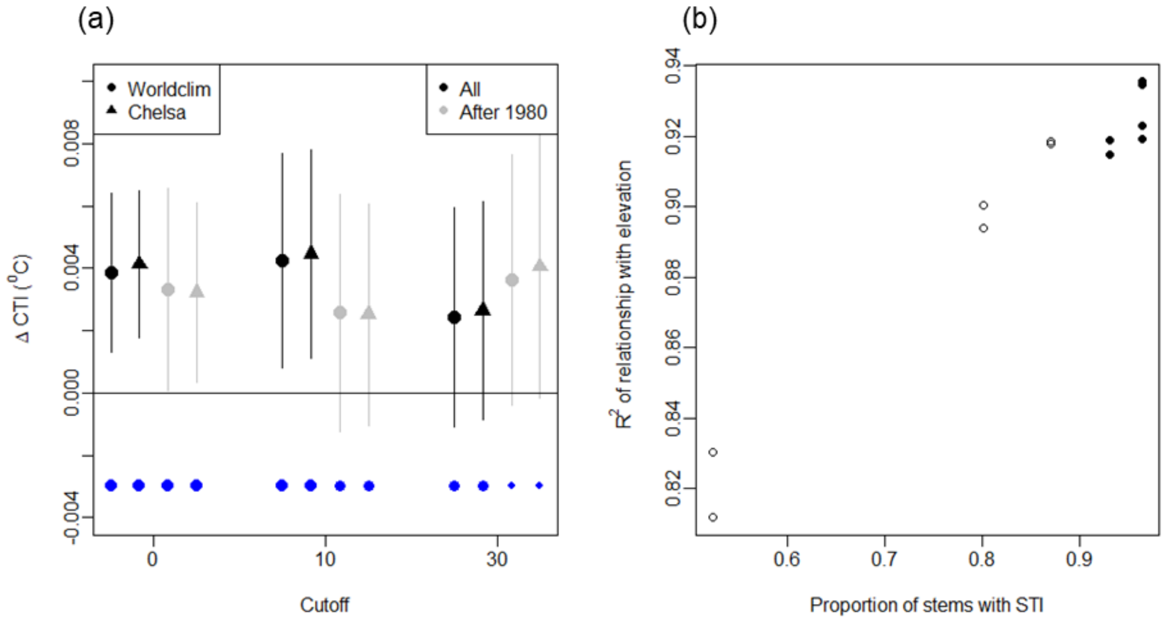

Supplementary Figure 1. (a) Effects of different criteria for i) excluding occurrence records (restricting species with fewer than a set number of records, or restricting records to those after 1980), or ii) using different sources of climate data, on estimated changes in the stem abundance weighted community thermal index (CTI_{Stem}). Symbols show rates of change in CTI, with error bars showing the 95% confidence intervals (n = 17 independent sampling plots, rates of change and confidence intervals estimated using an intercept only linear model). Blue circles are proportional in size to the proportion of stems with species thermal optima or index (STI) under each set of criteria. (b) Relationship between the R^2 of a linear model relating CTI_{Stem} to elevation with the proportion of stems with STI data under each criteria (same cutoffs as Left figure). Filled circles indicate criteria which yielded statistically significant changes in CTI_{Stem}, and open circles indicate criteria which yielded non-significant changes in CTI_{Stem}. Source data are provided as a source data file.

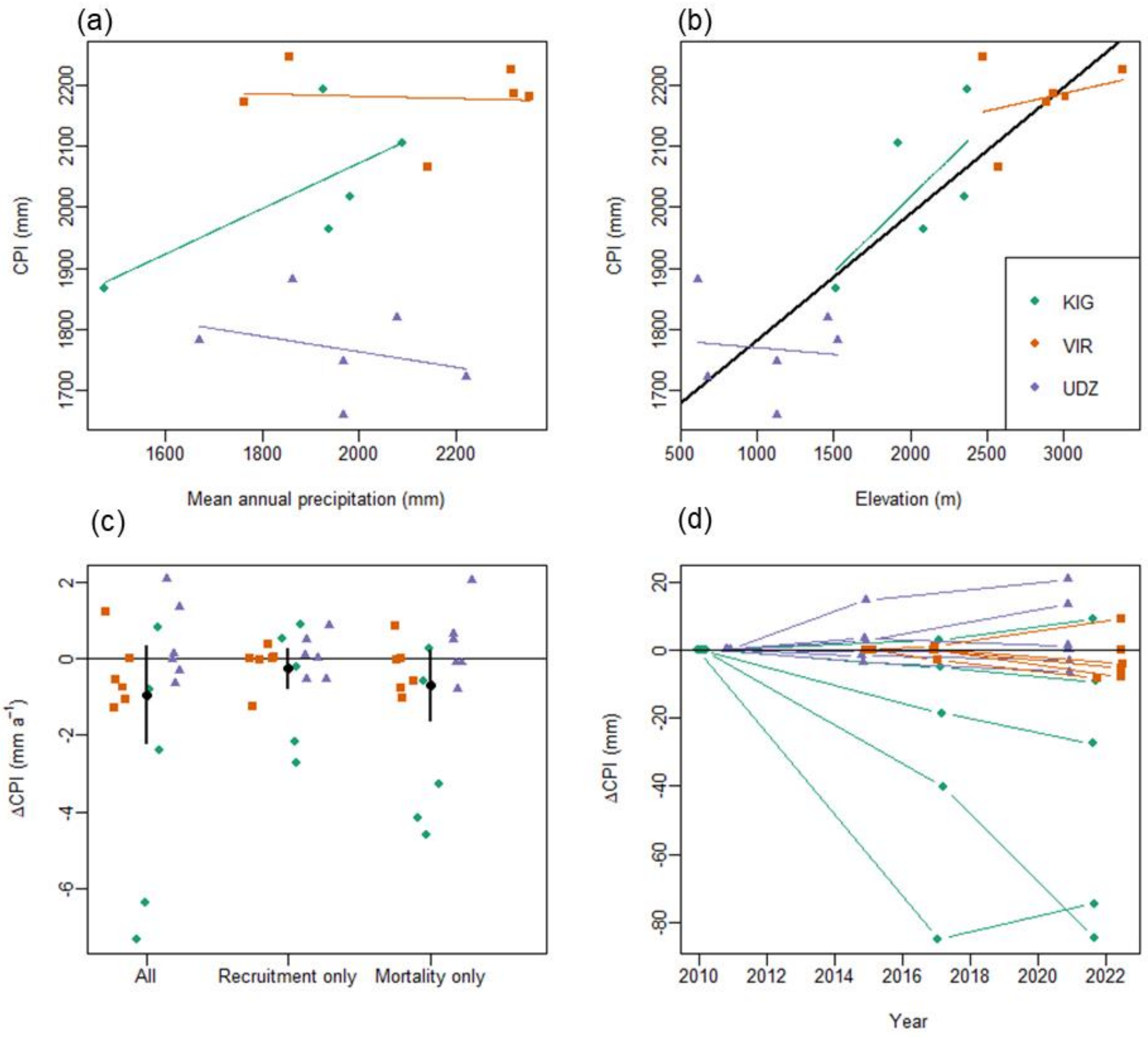

Supplementary Figure 2. (a) Relationship between stem abundance weighted community precipitation index (CPI) and mean annual precipitation. (b) Relationship between CPI and elevation. (c) Contribution of recruitment, mortality and growth to change in CPI. (d) Change in CPI between censuses at each plot. For a and b coloured lines show regression relationships within each site, and the black line shows the relationship across all sites. For c, points show changes for each plot, coloured by site. Black circles show average changes across plots, with error bars indicating 95% confidence intervals of changes (n = 17 independent sampling plots). For d, changes are expressed relative to values in the first census. KIG: Kigezi Highlands in Uganda; VIR: Virunga Mountains in Rwanda-Uganda, UDZ: Udzungwa Mountains in Tanzania. Source data are provided as a source data file.

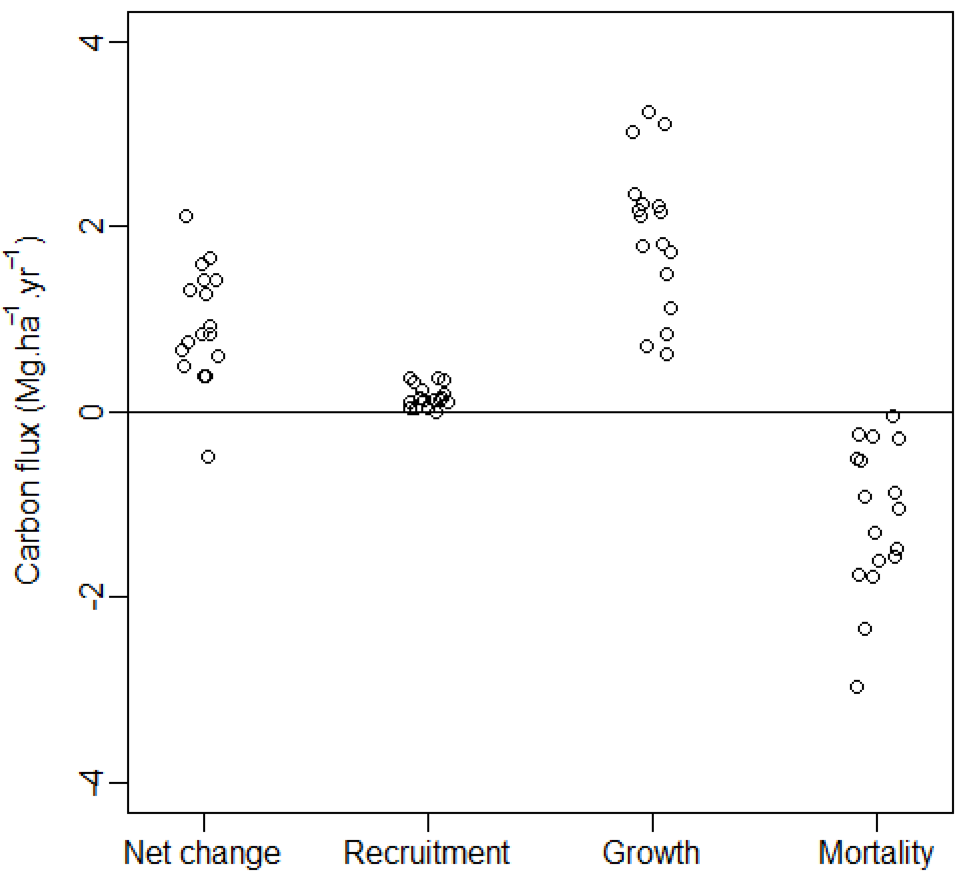

Supplementary Figure 3. Contribution of recruitment, growth and biomass mortality to net carbon fluxes. Carbon fluxes were not significantly related to thermophilization trends. Source data are provided as a source data file.

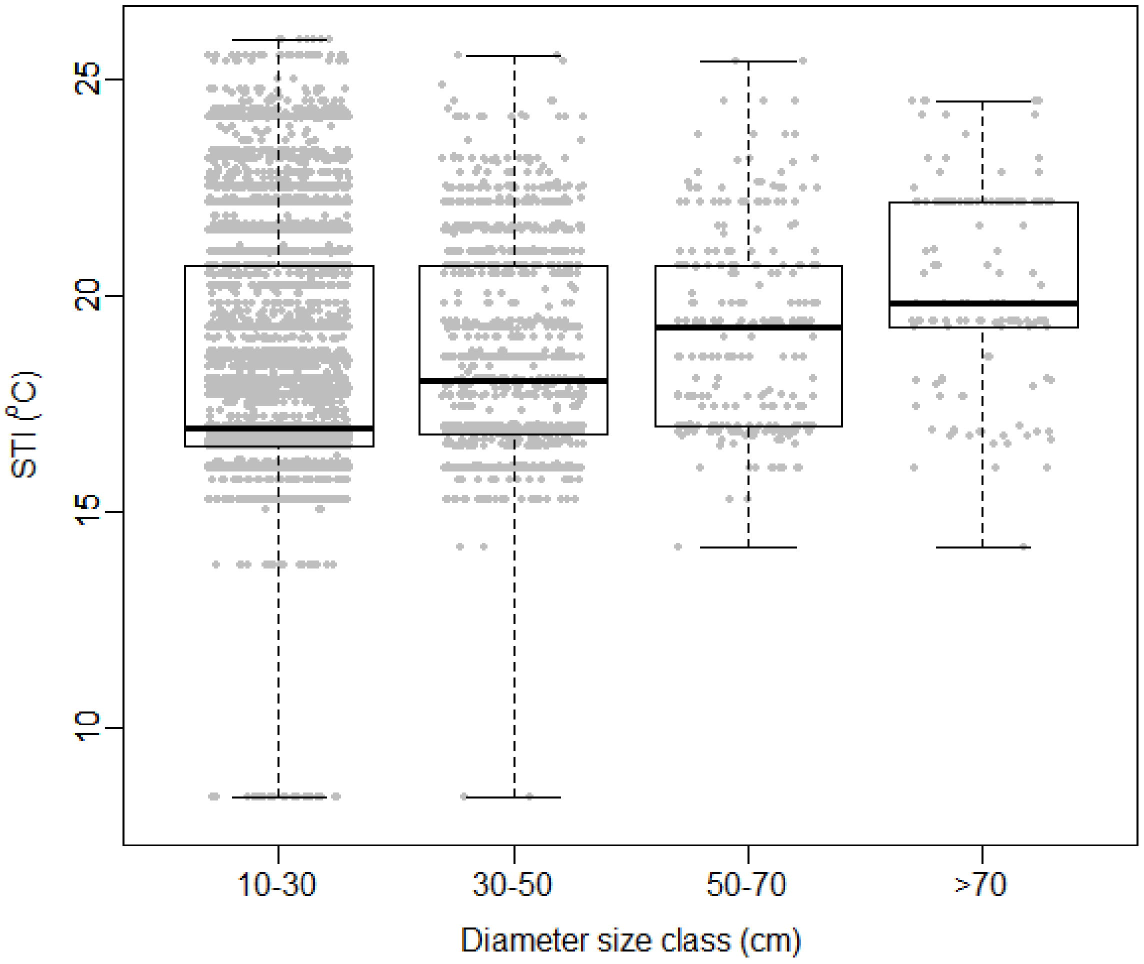

Supplementary Figure 4. Variation in species' thermal optima or index (STI) between size classes. The thermal optima of stems differed significantly between size classes, with stems in the >70cm diameter size class on average having a higher thermal optimum than stems in the 10-30cm diameter size class (mixed effect model with plot as a random effect, only using census one data, calculating P values with Satterthwaite's method, $t = 4.0$, $df=8108.4$, $P < 0.001$, conducted using R packages lme4 [51] and lmerTest [52]). N = 8128 trees in 17 plots. Source data are provided as a source data file.

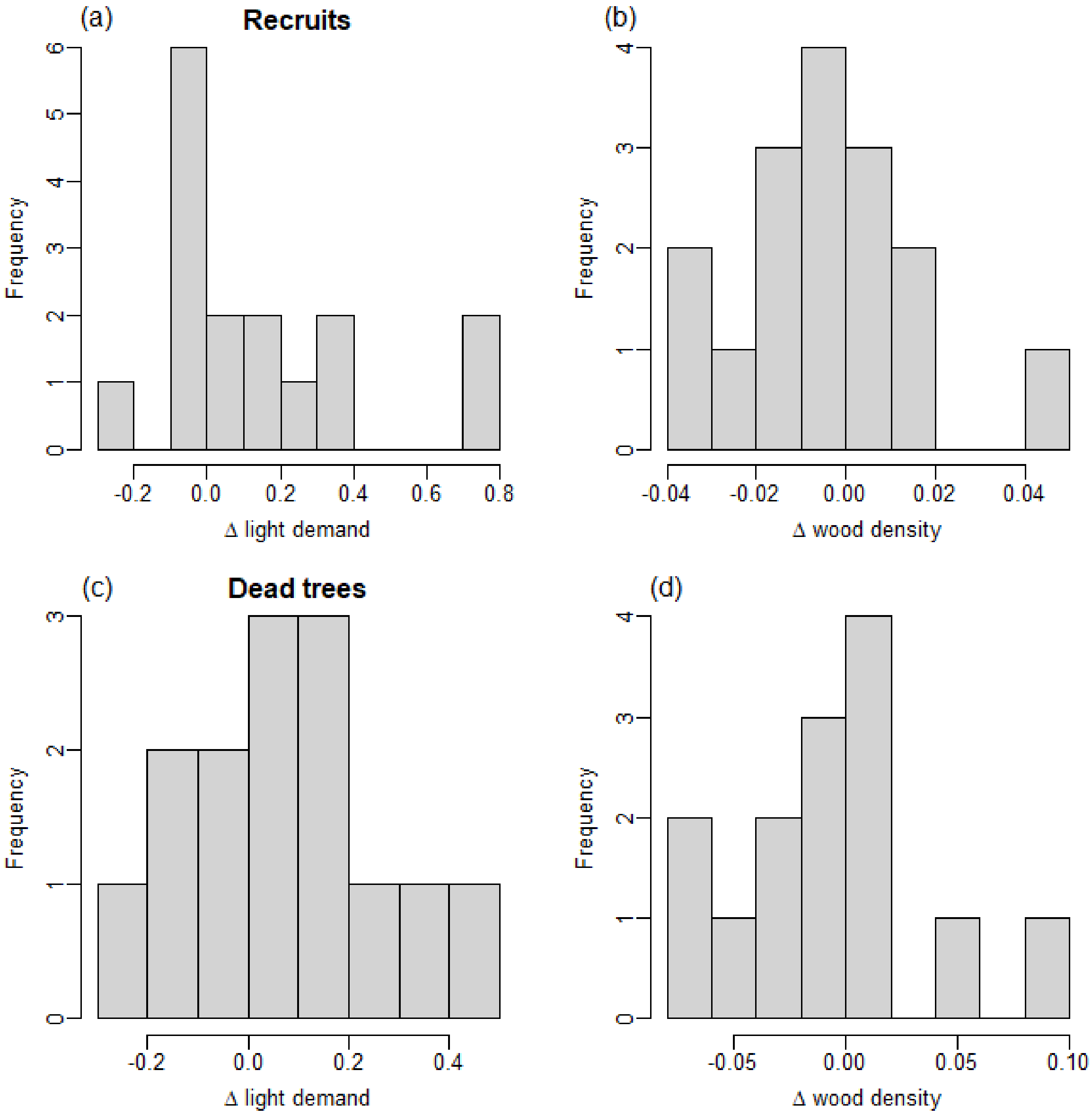

Supplementary Figure 5. Change in community weighted light requirements (higher values indicate greater dominance of shade bearers) and wood density in recruits (a,c) and in dead trees (b,d) between the second and third census. Positive values would indicate increasing dominance of shade bearers and species with higher wood density, expected with increasing succession. Community weighted light requirement score increased, but not significantly, for recruits (Wilcoxon signed-rank test, V = 74, n = 17, P = 0.050), providing some support for a shift in recruits towards more shade-bearing species, but changes in other variables were non-significant (wood density and recruits, V = 52, P = 0.433; light requirement and mortality, V = 64, P = 0.208; wood density and mortality, V = 43, P = 0.583). Source data are provided as a source data file.

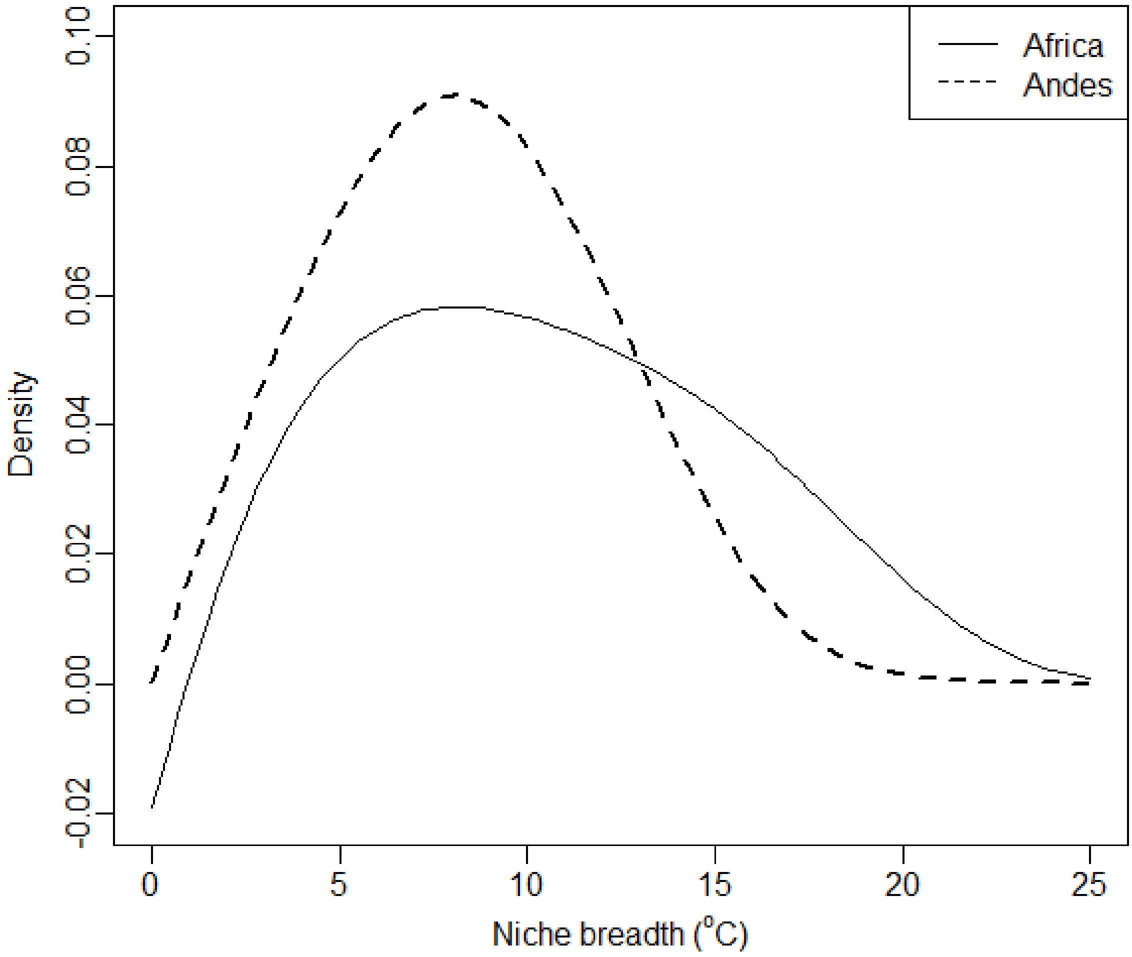

Supplementary Figure 6. Comparison of niche breath (difference between 0.05 and 0.95 quantile of mean annual temperature in locations species were recorded at) between our African dataset (n = 166 species) and a database of Andean tree species (n = 1293 species) records compiled by Fadrique et al. [7]. Probability density estimates were produced using kernel estimators for bounded data [49] in the bde R package [50]; note that the boundary kernel method used can produce negative density values. For species with ten or more records, niche breadth was broader for African trees (Africa median = 8.8°C, Andes median = 8.1°C, Wilcoxon test W=12275, P = 0.003). Source data are provided as a source data file.

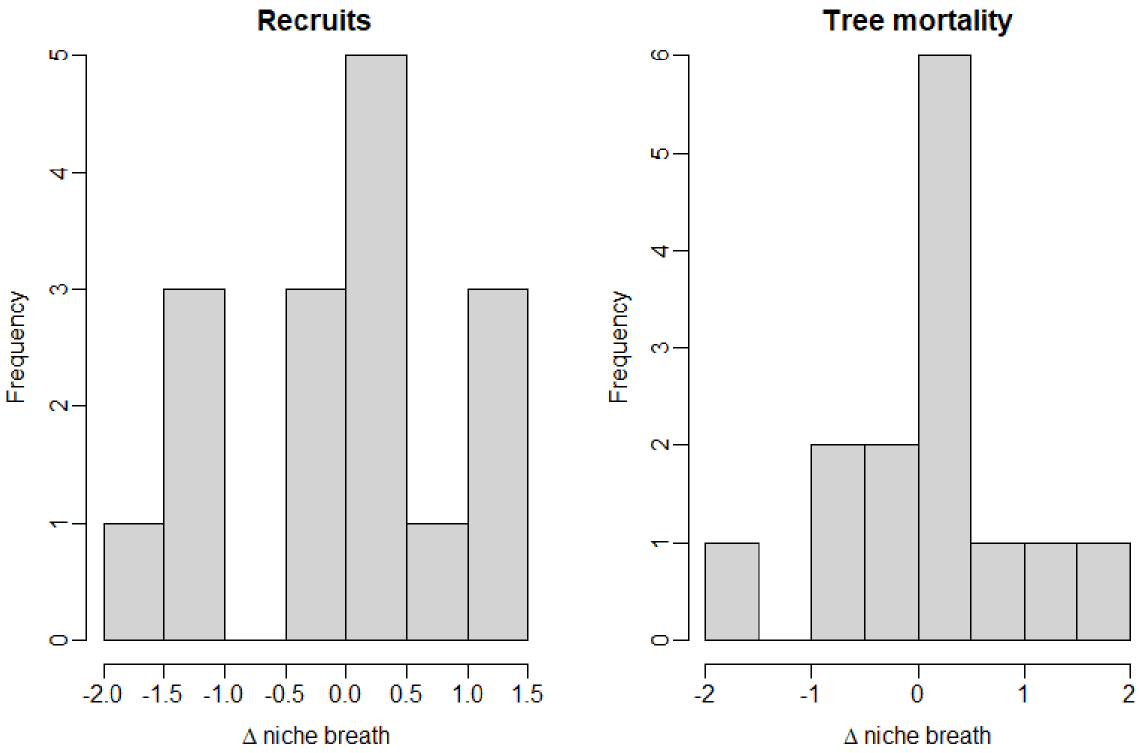

Supplementary Figure 7. Change in community weighted niche breadth (difference between 0.05 and 0.95 quantile of mean annual temperature in locations species were recorded at, higher values indicate increasing dominance of species with larger niche breadths in terms of mean annual temperature) in recruits (left) and dead trees (right) between the first and third census. Changes for both recruits and dead trees were non-significant (n = 17, recruits: V = 70, P = 0.934, mortality: V = 64, P = 0.502). Source data are provided as a source data file.

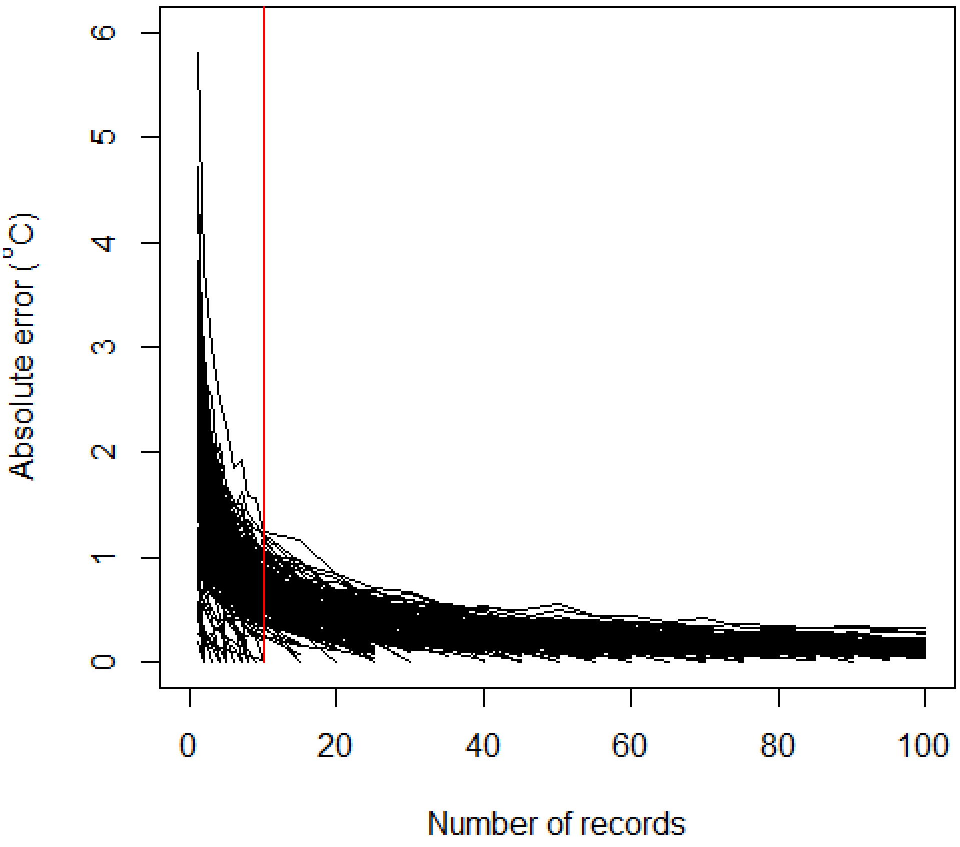

Supplementary Figure 8. Relationship between error in the estimation of a species' thermal optima or index (STI) and the number of occurrence records. Lines show the mean absolute error in STI across 100 random subsamples of occurrence records for each sample size. The red line shows the selected threshold of ten occurrence records. Source data are provided as a source data file.

Supplementary Table 1. Plot characteristics, dynamics and thermophilization rates. Community temperature index (CTI) is weighted by number of individuals (CTI_{Stem}) or by the basal-area of each stem (CTI_{BA}). CPI refers to community precipitation index weighted by number of individuals.

Plot	Elevation (m)	Stems identified to species (%)	Stem ha^{-1}	Species richness	Stem turnover (%)	Carbon stocks (Mg ha^{-1})	Carbon gains - growth (Mg ha^{-1} yr^{-1})	Carbon gains - recruits (Mg ha^{-1} yr^{-1})	Carbon loss - mortality (Mg ha^{-1} yr^{-1})	Net carbon change (Mg ha^{-1} yr^{-1})	ΔCTI_{Stem} (°C yr^{-1})	ΔCTI_{BA} (°C yr^{-1})	ΔCPI (mm yr^{-1})
UDZ-05	610	93.3	447	19	1.5	108.8	3.2	0.2	1.3	2.1	0.0019	0.0005	2.1
UDZ-03	670	98.8	450	20	1.1	214.1	3.1	0.1	1.6	1.7	0.0035	0.0044	0.1
UDZ-01	1124	89.4	454	32	1.7	312.7	3.1	0.3	3.0	0.4	-0.0008	-0.0092	-0.3
UDZ-02	1124	93.4	538	35	0.9	232.1	2.2	0.1	1.5	0.8	0.0037	0.0059	-0.7
UDZ-04	1456	86.5	686	45	0.7	265.2	1.8	0.3	0.9	1.3	0.0003	-0.0027	-0.6
UDZ-06	1523	85.7	599	33	0.6	244	2.1	0.1	0.9	1.3	0.0051	0.0009	-0.7
Udzungwa	1084.5	91.2	529.0	30.7	1.1	229.5	2.6	0.2	1.5	1.3	0.0023	0.0000	0.43
KIG-02	1511	95.3	555	47	1.7	119.9	2.2	0.2	1.8	0.7	0.0117	0.0139	-2.4
KIG-03	1921	99.3	603	39	1.7	100.2	2.2	0.4	1.8	0.8	0.0043	-0.0081	0.8
KIG-01	2084	100	278	30	2.4	131.1	1.8	0.1	0.5	1.4	-0.0010	-0.0029	-6.4
KIG-04	2353	99.3	669	32	0.8	190.1	2.4	0.1	1.0	1.4	0.0025	0.0065	-0.8
KIG-05	2374	99.9	536	29	4.0	75.3	1.5	0.4	2.3	-0.5	0.0254	0.0206	-7.3
Kigezi	2048.6	98.8	528.2	35.4	2.1	123.3	2.0	0.2	1.5	0.8	0.0086	0.0060	-3.21
VRU-04	2472	100	811	9	0.7	50.3	0.8	0.0	0.5	0.4	-0.0009	-0.0020	-0.6
VRU-05	2576	100	275	10	0.9	38.8	1.1	0.0	0.2	0.9	0.0031	0.0025	1.2
VRU-01	2891	100	668	12	1.4	88.5	2.2	0.1	1.6	0.8	0.0031	-0.0032	-1.3
VRU-06	2940	100	345	4	1.0	55.1	1.7	0.1	0.3	1.6	0.0024	0.0006	-0.7
VRU-02	3017	100	151	1	0.2	28.9	0.6	0.0	0.0	0.6	0.0000	0.0000	0.0
VRU-03	3388	100	446	2	1.0	24.3	0.7	0.0	0.3	0.5	0.0114	0.0058	-1.1
Virunga	2880.7	100.0	449.4	6.3	0.8	47.7	1.2	0.1	0.5	0.8	0.0032	0.0006	-0.41
All data	2002.0	96.5	500.6	23.5	1.3	134.1	1.9	0.2	1.1	1.0	0.0045	0.0020	-0.94

Supplementary Table 2. Relationship between carbon fluxes due to recruitment, growth and mortality and community temperature index (CTI) is weighted by stem abundance (CTI_{Stem}) or by the basal-area of each stem (CTI_{BA}). Correlation coefficients and (two-sided) P-values obtain from Spearman's rank correlation tests are shown. P values have not been adjusted for multiple comparisons.

	**CTI_{Stem}**	**CTI_{BA}**
Overall	r_s = -0.157, P = 0.547	r_s = -0.002, P = 0.996
Recruitment	r_s = 0.140, P = 0.592	r_s = -0.027, P = 0.921
Growth	NA	r_s = 0.201, P = 0.438
Mortality	r_s = 0.203, P = 0.432	r_s = 0.088, P = 0.737

Supplementary Table 3. Relationship between species' characteristics and their contribution to change in abundance weighted community thermal index (CTI). Coefficients are shown from the averaged linear model model following all-subsets regression (model-averaging heading), and from bivariate regression models (bivariate heading). P values are two-sided. Species' light requirements were classified into 0: no data, 1: pioneer light demander, 2: non-pioneer light demander, 3: shade bearer (see Methods). WD: wood density, STI: species thermal optima or index, SPI: species precipitation optima or index.

	Model-averaging				Bivariate			
	Estimate	SE	Z	P	Estimate	SE	t	P
(Intercept – Light 1)	-0.510	1.238	0.412	0.680	Varies between models			
Change	-0.138	0.209	0.662	0.508	-0.234	0.210	1.117	0.266
Height_max	0.013	0.012	1.062	0.288	0.016	0.009	1.833	0.069
WD	1.145	1.180	0.970	0.332	1.537	0.922	1.666	0.098
ln(Abundance)	0.034	0.066	0.509	0.611	0.115	0.074	1.552	0.123
STI	-0.010	0.029	0.351	0.725	-0.030	0.038	0.787	0.432
SPI	0.000	0.000	0.504	0.614	0.000	0.000	1.168	0.245
Light2	-0.011	0.122	0.092	0.927	-0.056	0.320	0.176	0.861
Light3	0.012	0.111	0.105	0.916	0.059	0.285	0.206	0.837

Supplementary Table 4. Correlates of change in abundance weighted community thermal index (CTI_{Stem}). Spearman's rank correlation coefficients and associated two-sided P values are shown for correlations of each variable with change in CTI.

Variable	r_s	P
Elevation	0.016	0.952
Percentage stems identified to species	-0.169	0.517
Species richness	0.261	0.311
Stem density	0.088	0.737
Stem turnover	0.203	0.432
Carbon stocks	-0.096	0.716
Carbon gains	0.073	0.782
Carbon sink	-0.188	0.469

Supplementary Table 5. Effect of removing individual plots on estimated change in CTI_{Sten}. Intercepts and P values are shown from intercept-only linear models constructed using all data except for the given plot.

Plot removed	ΔCTI_{Stem}	P
BWI-01	0.0048	0.011
VRU-04	0.0048	0.011
UDZ-01	0.0048	0.011
VRU-02	0.0047	0.012
UDZ-04	0.0047	0.013
UDZ-05	0.0046	0.015
VRU-06	0.0046	0.016
BWI-04	0.0046	0.016
VRU-01	0.0045	0.017
VRU-05	0.0045	0.017
UDZ-03	0.0045	0.017
UDZ-02	0.0045	0.017
BWI-03	0.0045	0.018
UDZ-06	0.0044	0.019
VRU-03	0.0040	0.026
BWI-02	0.0040	0.026
BWI-05	0.0031	0.005
