# Supplementary material for: Evidence of thermophilization in Afromontane forests
Source: Nat Commun. 2024 Jul 10;15:5554. doi: 10.1038/s41467-024-48520-w (PMC11236992; doi:10.1038/s41467-024-48520-w)
Supplement: Supplementary file 3 — Reporting Summary [file 41467_2024_48520_MOESM3_ESM.pdf]

nature portfolio

Corresponding author(s): Aida Cuni-Sanchez

Last updated by author(s): Apr 26, 2024

Reporting Summary

Nature Portfolio wishes to improve the reproducibility of the work that we publish. This form provides structure for consistency and transparency in reporting. For further information on Nature Portfolio policies, see our Editorial Policies and the Editorial Policy Checklist.

Statistics

For all statistical analyses, confirm that the following items are present in the figure legend, table legend, main text, or Methods section.

n/a	Confirmed	
☐	☒	The exact sample size (n) for each experimental group/condition, given as a discrete number and unit of measurement
☐	☒	A statement on whether measurements were taken from distinct samples or whether the same sample was measured repeatedly
☐	☒	The statistical test(s) used AND whether they are one- or two-sided<br>*Only common tests should be described solely by name; describe more complex techniques in the Methods section.*
☐	☒	A description of all covariates tested
☐	☒	A description of any assumptions or corrections, such as tests of normality and adjustment for multiple comparisons
☐	☒	A full description of the statistical parameters including central tendency (e.g. means) or other basic estimates (e.g. regression coefficient) AND variation (e.g. standard deviation) or associated estimates of uncertainty (e.g. confidence intervals)
☐	☒	For null hypothesis testing, the test statistic (e.g. F, t, r) with confidence intervals, effect sizes, degrees of freedom and P value noted<br>*Give P values as exact values whenever suitable.*
☒	☐	For Bayesian analysis, information on the choice of priors and Markov chain Monte Carlo settings
☒	☐	For hierarchical and complex designs, identification of the appropriate level for tests and full reporting of outcomes
☒	☐	Estimates of effect sizes (e.g. Cohen's d, Pearson's r), indicating how they were calculated

Our web collection on statistics for biologists contains articles on many of the points above.

Software and code

Policy information about availability of computer code

Data collection	No software was used.
Data analysis	We performed all analyses in R v.4.2.2 , and we used the MuMIn R package, bde R package, lme4 R package, lmerTest R package, the BIOMASS R package and the BiomasaFP R package

For manuscripts utilizing custom algorithms or software that are central to the research but not yet described in published literature, software must be made available to editors and reviewers. We strongly encourage code deposition in a community repository (e.g. GitHub). See the Nature Portfolio guidelines for submitting code & software for further information.

Data

Policy information about availability of data

All manuscripts must include a data availability statement. This statement should provide the following information, where applicable:

- Accession codes, unique identifiers, or web links for publicly available datasets
- A description of any restrictions on data availability
- For clinical datasets or third party data, please ensure that the statement adheres to our policy

Source data for figures are provided with this paper. Data supporting the findings of this study are available as a data package in ForestPlots.net [https://doi.org/10.5521/forestplots.net/2024_2 . Climate data were obtained from Chelsa (https://chelsa-climate.org/) and Worldclim V2 (https://www.worldclim.com/version2). Species occurrence records were obtained from the GBIF data portal (https://www.gbif.org/), and trait data obtained from the African Plant Database (http://africanplantdatabase.ch) and the PROTA database (https://www.prota4u.org/).

Input data underlying statistical analyses are available at [https://doi.org/10.5521/forestplots.net/2024_2].
The R code used in this study is available as a data package in ForestPlots.net [https://doi.org/10.5521/forestplots.net/2024_2].

Research involving human participants, their data, or biological material

Policy information about studies with human participants or human data. See also policy information about sex, gender (identity/presentation), and sexual orientation and race, ethnicity and racism.

Reporting on sex and gender	N/A
Reporting on race, ethnicity, or other socially relevant groupings	N/A
Population characteristics	N/A
Recruitment	N/A
Ethics oversight	N/A

Note that full information on the approval of the study protocol must also be provided in the manuscript.

Field-specific reporting

Please select the one below that is the best fit for your research. If you are not sure, read the appropriate sections before making your selection.

☐ Life sciences ☐ Behavioural & social sciences ☒ Ecological, evolutionary & environmental sciences

For a reference copy of the document with all sections, see nature.com/documents/nr-reporting-summary-flat.pdf

Ecological, evolutionary & environmental sciences study design

All studies must disclose on these points even when the disclosure is negative.

Study description	We sampled 17 1-hectare inventory plots located between 610 and 3,388 m asl in 3 different mountains, at three points in time between 2010 and 2022.
Research sample	17 forest plots of 1 hectare each, these were the only permanent plots available in the mountains studied.
Sampling strategy	These were the only permanent plots available in the mountains studied. Previous work on thermophilization of Andean forests used a similar sample size
Data collection	In all plots and census, tree diameter of all stems ≥10cm diameter was measured at 1.3 m along the stem from the ground (or above buttresses if present) and each stem was identified to species. In the last census, tree height (measured using a handheld laser Nikon Forestry Pro) was also recorded for about 60 stems per plot comprising several individuals from each diameter class. Fieldwork in Uganda was led by co-author A.N. in all censuses, in Tanzania by E.H.M. and in Rwanda by E.U.
Timing and spatial scale	All plots studied were sampled three times between 2010 and 2022.The timing of these 3 census varies between mountains as it was dependent on funding of different projects, and availability of different field teams.
Data exclusions	No data was excluded from the analysis
Reproducibility	These are observational measurements of dynamic natural forests (not plantations or experiments). As trees continue to grow, die and recruit, our measurements are time-specific but as our methods are standard, the methods could be applied to other mountain forests.
Randomization	In the first census, plots were established along elevation gradients within a mountain, also based on accessibility for field teams given complex terrain found in these mountain areas. We sampled all permanent old-growth forest plots available in the mountains studied.
Blinding	Not applicable, we sampled all permanent forest plots available in the mountains studied.

Did the study involve field work? ☒ Yes ☐ No

Field work, collection and transport

Field conditions	See supplementary Table 1.

Location	Bwindi National Park in the Kigezi Highlands (Uganda), Volcanos National Park in the Virunga Mountains (Rwanda) and Udzungwa National Park in Udzungwa Mountains (Tanzania) (Fig. 1).
Access & import/export	No plant samples were collected and exported for this research. Uganda: in August 2021 a research permit was obtained from the Uganda Wildlife Authority (COD 9605) and from the Uganda National Council for Science and Technology (number NS282ES). Tanzania: In February 2022 a research permit was obtained from the Tanzania Commission for Sciences and Technology (number 2021-104-NA-2021-212). Rwanda: the Rwanda Development Board (RDB) Tourism & Conservation granted permission to E.H. Martin for carrying out this research in Volcanos National Park in May 2022 (no specific permit number issued).
Disturbance	No disturbance to the forests was caused by this study as we used allometric equations to estimate carbon.

Reporting for specific materials, systems and methods

We require information from authors about some types of materials, experimental systems and methods used in many studies. Here, indicate whether each material, system or method listed is relevant to your study. If you are not sure if a list item applies to your research, read the appropriate section before selecting a response.

Materials & experimental systems

n/a	Involved in the study
☒	☐ Antibodies
☒	☐ Eukaryotic cell lines
☒	☐ Palaeontology and archaeology
☒	☐ Animals and other organisms
☒	☐ Clinical data
☒	☐ Dual use research of concern
☒	☐ Plants

Methods

n/a	Involved in the study
☒	☐ ChIP-seq
☒	☐ Flow cytometry
☒	☐ MRI-based neuroimaging

Plants

Seed stocks	N/A
Novel plant genotypes	N/A
Authentication	N/A
